# A systematic review to investigate whether birth weight affects the autonomic nervous system in adulthood

**DOI:** 10.1590/1984-0462/2024/42/2023002

**Published:** 2023-11-03

**Authors:** Giovanna de Paula Vidigal, Luana Almeida Gonzaga, Andrey Alves Porto, David Matthew Garner, Vinicius Ferreira Cardoso, Vitor Engrácia Valenti

**Affiliations:** a Universidade Estadual Paulista, Presidente Prudente, SP, Brazil.; b Oxford Brookes University, Oxford, United Kingdom.

**Keywords:** Autonomic nervous system, Birth weight, Cardiovascular diseases, Cardiovascular system, Heart rate, Sistema nervoso autônomo, Peso ao nascer, Doenças cardiovasculares, Sistema cardiovascular, Frequência cardíaca

## Abstract

**Objective:**

To evaluate the relationship between birth weight and the autonomic nervous system in adulthood through a systematic review.

**Data source:**

This is a systematic review of publications without limitation of year and language. We included studies involving the autonomic nervous system and birth weight in adults. Manuscripts were selected based on electronic searches of Medical Literature Analysis and Retrieval System Online (MEDLINE), Cumulative Index to Nursing and Allied Health Literature (CINAHL), Web of Science Cochrane Library and Scopus databases, using “Autonomic Nervous System” OR “Heart Rate” OR “Heart Rate Variability” AND “Birth Weight” as a search strategy. This review is registered on the International Prospective Register of Systematic Reviews — PROSPERO (ID: CRD42020165622).

**Data synthesis:**

We found 894 articles; 215 were excluded for duplicity. Of the remaining 679 studies, 11 remained. Two were excluded because they did not specifically treat the autonomic nervous system or birth weight. There were nine publications, two cohort and seven cross-sectional studies. The main findings were that extreme, very low, low or high birth weight may have some impact on the autonomic nervous system in adult life.

**Conclusions:**

Birth weight outside the normality rate may have a negative influence on the autonomic nervous system, causing autonomic dysfunction and increasing the risk of cardiovascular diseases in adult life. Thus, the importance of the follow-up of health professionals from pregnancy to gestation and throughout life, with preventive care being emphasized.

## RESUMO

**Objetivo:**

Avaliar a relação entre o peso ao nascer e o sistema nervoso autônomo na vida adulta por meio de uma revisão sistemática.

**Fontes de dados:**

Esta é uma revisão sistemática de publicações, sem limitação de ano e idioma. Incluímos estudos envolvendo o sistema nervoso autônomo e peso ao nascer em adultos. Os manuscritos foram selecionados das bases de dados eletrônicos Medical Literature Analysis and Retrieval System Online (MEDLINE), Cumulative Index to Nursing and Allied Health Literature (CINAHL), Web of Science Cochrane Library e Scopus, utilizando “Autonomic Nervous System” OR “Heart Rate” OR “Heart Rate Variability” AND “Birth Weight” como estratégia de busca. Esta revisão está registrada pelo International Prospective Register of Systematic Reviews — PROSPERO (ID: CRD42020165622).

**Síntese dos dados:**

Nós encontramos 894 artigos. Deles, 215 foram excluídos por duplicidade. Entre os 679 remanescentes, 11 permaneceram, dos quais dois foram excluídos por não tratarem especificamente do sistema nervoso autônomo ou do peso ao nascer. Restaram nove publicações, sendo duas longitudinais e sete transversais. Os principais achados foram que o peso extremo baixo, muito baixo, baixo ou alto ao nascer pode ter algum impacto no sistema nervoso autônomo na vida adulta.

**Conclusões:**

O peso ao nascer fora da normalidade pode influenciar negativamente o sistema nervoso autônomo, causando disfunção autonômica e aumentando o risco de doenças cardiovasculares na vida adulta. Assim, ressalta-se a importância do acompanhamento dos profissionais de saúde desde a gravidez até a gestação, pré-natal e ao longo da vida, com cuidados preventivos para esta situação.

## INTRODUCTION

Cardiovascular diseases (CVD) are the leading cause of mortality in the world, both in developed and developing countries.^1^ According to data from the World Health Organization (WHO), the leading causes of death worldwide in the last 15 years are ischemic heart disease and stroke.^2^

There are several reasons that may contribute to CVD, which may be due to lifestyle (physical inactivity, poor diet, alcohol and/or tobacco consumption, overweight, obesity and others) or health factors (family history/genetics, high cholesterol, high blood pressure), diabetes *mellitus*, metabolic syndrome and chronic kidney disease.^3^

Increased activity of the sympathetic autonomic nervous system (SANS) and a reduced parasympathetic nervous system activity induce CVD. Thus, the organism has a higher energy demand, causing an autonomic imbalance.^4^

Adults who were born preterm, regardless of birth size, were at higher risk for developing CVD.^5^ This is because the maturation of the nervous system begins in the third trimester of pregnancy, according to Gagnon et al.,^6^ and continues to develop after birth.^7^ Before 37 completed weeks, autonomic nervous system (ANS) maturation is temporarily interrupted. Consequently, autonomic regulation of heart rate (HR) is impaired, leading to damage in childhood and even in adulthood.^8-10^

As evidenced in the systematic review by Cardoso et al.,^11^ preterm infants have lower parasympathetic activity than full-term infants. Many articles address this type of population; however, little is known about birth weight (BW) specifically.^11^

Therefore, we question: is there a relationship between BW and ANS in adult life? Does this group have a higher cardiovascular risk? Our intention is to alert and promote prevention. Although there are studies that suggest that cardiovascular regulation may be altered according to birth weight, relatively little is known whether these changes in the ANS persist into adulthood. We tested whether BW predicts measures of autonomic regulation at rest. Thus, we aimed to evaluate the relationship between body weight and ANS in adulthood through a systematic review.

## METHOD

This systematic review was conducted between September 2017 and June 2019, following the Preferred Reporting Items for Systematic Reviews and Meta-Analyzes (PRISMA) protocol. This review is registered on the International Prospective Register of Systematic Review — PROSPERO (ID: CRD42020165622).

No language limitation, year of publication and study design was applied. The search and selection of studies were performed using the strategy Population (P), Exposition (E), Outcome (O) (PEO), an acronym used to delimit what studies would be considered for final revision. In this research, human adults were defined as the “population”, birth weight as interest “exposition”, and ANS-related issues as “outcome”. After defining the components, keywords were searched using the Medical Subject Headings (MeSH). The search for articles in the literature was done through electronic databases Medical Literature Analysis and Retrieval System Online (MEDLINE), Cumulative Index to Nursing and Allied Health Literature (CINAHL), Web of Science Cochrane Library and Scopus. The search strategy used was “Autonomic Nervous System” OR “Heart Rate” OR “Heart Rate Variability” AND “Birth Weight” and their derivatives, both in title or abstract. A search was also made in the P@rthenon database of the São Paulo State University.

The database was exported to EndNote (Clarivate Analytics, USA). After data export, duplicate studies were excluded. Then, a title and abstract screening was performed that obeyed the inclusion criteria. The texts were read in full by two researchers independently and, if there was discrepancy regarding the inclusion or not of a certain article, an extra reviewer was consulted to make a final choice.

The methodological quality of the articles was assessed through the National Heart, Lung, and Blood Institute (NHLBI) Quality Assessment Tool for Observational Cohort and Cross-Sectional Studies, National Institute of Health (NIH), to analyze the strength of the evidence of the articles.^12^

The questionnaire consisted of 14 questions about the publication, such as clarity of the objective, dependent and independent variables, the sample, and whether there was more than one evaluation during the research time. The answers are: “Yes”, “No” and “Other (cannot determine; not applicable; not reportable)”.^12^

This questionnaire is not a checklist to mark answers according to each question, it calculates and provides the quality of the article. It is a form intended to help focus on key concepts and thus consider risk of bias for the researchers’ analysis. With this, two evaluators independently answered the questions and rated them as “good”, “fair” or “bad”. If there was a discrepancy regarding the inclusion of an article, an extra reviewer was consulted for the final classification.

Some criteria were followed for choosing the articles that would be included in this systematic review. Firstly, we analyzed whether the article had evaluated heart rate variability (HRV) or HR or ANS-related outcomes, then if there was information on birth weight, since many studies reported only age or size at birth.

The chosen population over five years old. Studies that were only with newborns or that were not related to the theme were not selected. Study designs that specifically dealt with birth weight or ANS were selected. The articles chosen had cross-sectional or cohort study designs. Review papers were not included.

## RESULTS

Initially, 894 articles were found and 215 were excluded for duplication, resulting in 679. Then, the screening of titles and abstracts resulted in 11 studies that met the inclusion criteria. All were read in full and, of them, two were excluded because they did not specifically deal with BW or ANS. The final studies were presented in two tables for better visualization and organization. Table 1 contains: author (year), follow-up period, final sample and main conclusions. Tables 2 and 3 show the general characterization of the studies (from 1997 to 2009 and from 2010 onward, respectively), indicating author (year), collected data, protocol, conclusion (outcome) and quality score of each study.

**Table 1:** Summary of selected studies investigating the association between birth weight and autonomic nervous system in adulthood.

Author	Follow up	Final sample	Main conclusions
Phillips & Barker^18^	No	449 subjects LBW and NBW 46-54 years and healthy	Raised blood pressure and insulin resistance may partly result from a primary increase in SNS activity that is initiated in utero and persists into adult life.
Weitz et al.^20^	No	26 subjects LBW and NBW 20-30 years and healthy	Findings do not suggest that changes in sympathetic outflow to the muscle vascular bed are responsible for the increased prevalence of hypertension in this group of subjects.
Ward et al.^16^	No	179 subjects LBW, NBW, HBW ~30 years	Cardiovascular responses to psychological stressors may be programmed antenatally and suggest a potential mechanism linking reduced fetal growth with raised blood pressure and cardiovascular disease in adulthood.
Jones et al.^15^	No	179 subjects LBW and NBW ~26 years	The study suggests that women and girls who were small at birth have greater sympathoadrenal activity than their higher birth weight peers, whereas men and boys who were small at birth have an enhanced adrenocortical response to stress, although this needs confirmation.
Mathewson et al.^14^	Yes. 35 years	77 subjects ELBW and NBW 22-35 years	RSA may also be less stable over time in some ELBW survivors than is generally the case for NBW controls, suggesting a decrement in parasympathetic regulatory control that may warrant closer monitoring as ELBW survivors age.
Perkiömäki et al.^17^	No	4078 subjects LBW, NBW, HBW 46 years and healthy	The findings suggest that greater, not depressed, prenatal growth may contribute to poorer cardiovascular autonomic regulation and the related cardiac risk in later life in men.
Bao et al.^19^	No	21 subjects LBW, NBW, HBW 23-24 years and healthy	The study suggests that LBW relates to increased low-grade inflammation and blunted autonomic function in healthy young Mongolian adults, and these might be preliminary steps of hypertension development in LBW individuals.
O'Hare et al.^13^	Yes. 6 decades	4799 subjects LBW and NBW 6 up to 69 years	Higher birth weight and conditional BMI change were associated with lower RHR at age 6 and across the life course.
Haraldsdottir et al.^21^	No	28 subjects VLBW and NBW ~26-29 years	The study demonstrates that HRR is significantly slower in healthy young adults born preterm compared to age-matched, term-born controls.

LBW: Low Birth Weight (1500–2500 g); NBW: Normal Birth Weight (2500g–4000 g); SNS: Sympathetic Nervous System; HBW: High Birth Weight (macrosomia> 4000 g); ELBW: Extreme Low Birth Weight (<1000 g); BMI: body mass index; VLBW: Very Low Birth Weight (1000–1500 g); RHR: Higher resting heart rate; HRR: Heart rate recovery.

**Table 2:** Description of references selected from 1997 up to 2009, according to author, data collected, protocol, conclusion and score.

Author	Collected data	Protocol	Conclusion (Outcome)	Score
Phillips & Barker^18^	Birth data; Anthropometric measurements; Blood collection and oral glucose tolerance test.	BP and HR measurements after 5 minutes of rest; interview about family history and quality of life; BP and HR measurements.	In addition to increased HR, increased BP and insulin resistance during adulthood with LBW may occur due to increased intrauterine SNS activity; risk of CVD.	Good
Weitz et al.^20^	Physical activity evaluation, laboratory screening, blood collection, anthropometric measurements, and 24-hour ambulatory BP measurement; electromyography of the superficial peroneal nerve; BP measurements, HR monitoring and respiratory.	Rest for 10 minutes with oscillometric BP measurement; performing maximum length inspiratory apnea; 10-minute recovery.	Low sympathetic activity in muscle vascular bed at basal conditions may be considered a primary biological marker in LBW; changes in sympathetic flow in muscular vascular bed are not responsible for the increase.	Good
Ward et al.^16^	Birth information; anthropometric measurements; health status and habits, and socioeconomic status; continuous monitoring of HR and BP.	Initial rest; three psychological tests, interspersed with a six-minute rest: I) color-word conflict task, II) mirror-tracing task, III) speech task.	Association between cardiovascular reactivity, psychological stressors and sizes at birth, leading to the hypothesis that these responses may be programmed in the prenatal period; increased BP and risk of CVD in adulthood.	Good
Jones et al.^15^	Birth information; information on state and current health habits and socioeconomic status; continuous BP and HR measurements; collection of saliva.	Initial rest of 20 minutes; three psychological tests, interspersed with a six-minute rest: I) stroop word-color conflict task, II) mirror-tracing task, III) speech task.	Impaired fetal growth, autonomic cardiovascular control and baroreflex function are related, and may be sex-dependent; possibility of SAH and later diseases.	Poor

BP: Blood Pressure; HR: Heart Rate; LBW: Low Birth Weight; SNS: Sympathetic Nervous System; CVD: Cardiovascular Disease.

**Table 3:** Description of references selected from 2010 onwards, according to author, data collected, protocol, conclusion and score.

Author	Collected data	Protocol	Conclusion (Outcome)	Score
Mathewson et al.^14^	EEG and ECG and ASR records at 22–26 years and 30–35 years.	ECG: Continuous recording for 2 minutes at rest in the sitting position; 30-35 years: continuous registration for 6 minutes; ASR registration for 5 minutes.	Altered parasympathetic regulation in adults with ELBW, which can early identify the risk of CVD.	Good
Perkiömäki et al.^17^	Health and lifestyle status questionnaires; examination of blood collection, the oral glucose tolerance test, BP, HR, RF, BRS.	3 minutes in sitting position; 3 minutes standing, with spontaneous breathing.	Higher prenatal growth may contribute to cardiovascular risk in middle-aged men; in women, of course.	Good
Bao et al.^19^	Questionnaire on BW, anthropometric data, quality of life, family history, blood collection; BP and HR measurements in lying, sitting and immediately after sitting positions.	15 minutes in supine position; Postural change; 15 minutes in sitting position	Increased low-grade inflammation and reduced autonomic function in LBW in adult Mongolians; May be determinant for development of SAH.	Good
O'Hare et al.^13^	Social, anthropometric and developmental records at birth and throughout life; resting HR and anthropometric measurements throughout life (for six decades). Maximal power, exhaustion time, maximum aerobic capacity, VO_2_max test.	HR measurement at rest in sitting position.	Critically high resting CF and increased middle age are not associated with CVD risk; Early life is essential in determining the course of resting HR until adulthood.	Fair
Haraldsdottir et al.^21^	Weight and height; Global Physical Activity Questionnaire; breath-to-breath; HR and ventilatory and metabolic parameters; maximal power, exhaustion time, maximum aerobic capacity, VO_2_max test, ventilatory threshold; HR recovery.	Two progressive exercise tests on vertical cycle ergometer: I) breathing normal air and II) breathing hypoxic air. Normal protocol until they are no longer able to maintain 55 rpm for more than 5s; rest of 45 minutes; inhalation of hypoxic gas; hypoxic protocol until maximum exhaustion.	Significantly slower HR recovery in healthy preterm young adults; lower aerobic fitness in these individuals; increased cardiovascular risk and mortality.	Good

EEG: electroencephalogram; ECG: electrocardiogram; ASR: respiratory sinus arrhythmia; ELBW: extreme low birth weight; CVD: cardiovascular disease; BP: blood pressure; HR: heart rate; RF: respiratory frequency; BRS: baroreflex sensitivity; BW: birth weight; LBW: low birth weight; SAH: systemic arterial hypertension; VO_2_max: maximal oxygen consumption.

**Figure 1:**
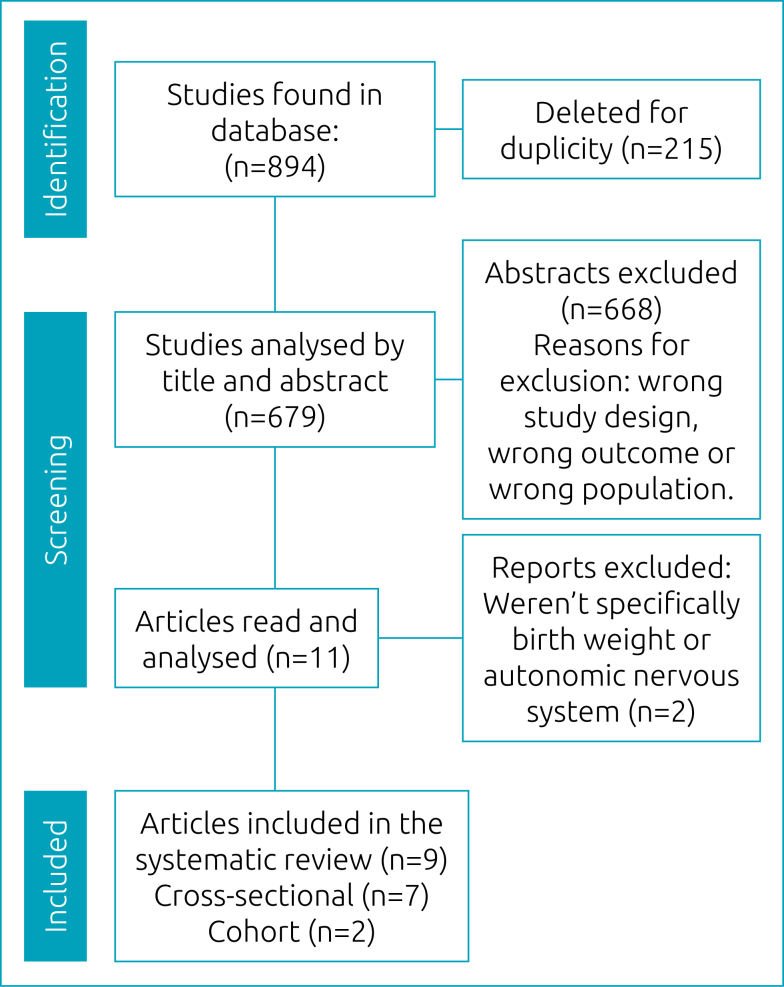
Search process results according to PRISMA flow diagram.

Nine final publications were selected for this review, two of which cohort^13,14^ and seven cross-sectional.^15-21^ The search process and study selection steps are presented in a flow diagram of the PRISMA protocol (Figure 1). The surveys were conducted on subjects based on BW and contained at least one of the groups: extremely low birth weight (ELBW), very low birth weight (VLBW), low birth weight (LBW), normal birth weight (NBW) and high birth weight (HBW). All had an association with the ANS.

In general, the purpose of the studies was to investigate whether there is a relationship between ANS and BW during adulthood. For this, HR was measured in all of them, and some measured other variables that could have an influence, such as blood pressure (BP) by Jones et al.,^15^ Ward et al.,^16^ Phillips & Barker et al.,^18^ Bao et al.,^19^ and Weitz et al.,^20^ psychological stress by Ward et al.^16^ and Jones et al.,^15^ muscle electrical activity by Weitz et al.,^20^ lung capacity by Haraldsdottir et al.,^21^ insulin resistance by Perkiömäki et al.,^17^ blood collection by Phillips & Barker et al.,^18^ Weitz et al.,^20^ Perkiömäki et al.^17^ and Bao et al.,^19^ baroreflex sensitivity by Perkiömäki et al.,^17^ among others.

Regarding methodological quality, assessed using the NHLBI, NIH Quality Assessment Tool for Observational Cohort and Cross-Sectional Studies,^12^ we observed the following results: five articles (77.7%) presented good quality, one (11.1%) had fair quality and one (11.1%) was poor. Table 2 shows the characteristics of each selected article regarding protocols, outcomes and scores.

All nine articles addressed HR. However, each one evaluated different parameters. O'Hare et al.^13^ only and specifically analyzed resting HR.

This study lasted about six decades, as it followed volunteers from birth, gathering gestational information, parental socioeconomic status (at four years old), breastfeeding age, cognitive factors such as reading comprehension, pronunciation, vocabulary and nonverbal reasoning (at eight years old) and lifestyle, such as smoking habits, height, weight and body mass index over the years. Resting HR measurements were taken at six, seven, 11, 36, 43, 53, 60 to 65 and 69 years old.

Subjects with LBW had a higher mean resting HR than NBW. The authors concluded that early life is important in determining the trajectory of resting HR throughout lifetime. Although resting HR was critically high and increased in middle age, there was no association between BW and CVD risk.

Despite clearly demonstrating the objective, population and study variables, there was no standardization of HR measurement over the years and the protocol was not well specified as a resting time before and between measurements. During childhood (six, seven and 11 years old) HR was obtained by radial palpation twice during a physical examination at school by a physician; the second measure was used for analysis. At 36- and 43-years old HR was also counted by radial palpation only one time by nurses. At 53, 60 to 64 and 69 years old, HR count was performed by an automatic BP device, with three measurements (except at 53 years old, with only two). For analysis, the first or the second measurement was used. Thus, the methodological quality of this article was fair, since different methodologies may influence the results.

Two articles evaluated autonomic function. The study conducted by Perkiömäki et al.^17^ performed examinations in middle-aged men and women separately. The authors analyzed autonomic function (HR, BP and respiratory frequency), baroreflex sensitivity and blood samples. These variables were evaluated in the sitting position and orthostatism, with spontaneous breathing, for three minutes each. The authors found that vagal activity and baroreflex sensitivity were lower in men born with HBW than in NBW and LBW. In women, the findings were unclear.

Bao et al.^19^ studied the relationship between LBW and autonomic function in Mongolian young adults (men and women 23–24 years), since Mongolians are known to be hypertensive, as well as individuals with LBW. They were compared with NBW. Blood was collected to analyze the inflammatory markers, as well as a 15-minute decubitus protocol followed by 15 minutes at sitting position, in which HR and BP were investigated. It was concluded that there was an increase in low-grade inflammation as well as impaired (reduced) autonomic function in young Mongolians with LBW.

Both articles had methodological quality considered “good”, since the objectives, measures and analyses were clearly described, and there were no major losses during the evaluations and analyses.

Only one article has examined the relationship between LBW, insulin resistance and BP. In the study conducted by Phillips & Barker et al.,^18^ this relationship in men and women aged 46 to 54 years was examined. LBW was compared with NBW. The 5-minute sitting protocol, questionnaire, and final HR and BP measurements indicated not only increased HR, but also increased BP and insulin resistance, suggesting that it may be due to increased intrauterine SANS activity. The lower the BW, the higher the resting HR. Methodological quality was considered good.

The article published by Weitz et al.^20^ compared muscle sympathetic nerve activity (MSNA) in 20 to 30-year olds of both sexes, since dysfunction in this nerve may contribute to the development of systemic arterial hypertension (SAH) and obesity in people with LBW. They were compared with NBW.

Then, the traffic of this nerve was examined at rest, as well as its modulation of the baroreflex in the vascular bed of the muscle in the following protocol: ten minutes of rest, maximum length respiratory apnea and ten minutes of recovery. Blood, HR, BP, respiratory movements and electromyography were collected, as well as vasoactive drugs administration.

As a result, MSNA was significantly lower in resting LBW, and significantly higher during apnea. It was pointed out that their baroreflex-mediated alterations in the experimental alterations of BP were not altered in the subjects with LBW. It would also need to determine the functional consequences of this activity.

It was argued that low ANS under baseline conditions may be considered a biological landmark in LBW, but despite this, these changes in sympathetic flow of the muscular vascular bed are not responsible for the prevalence of hypertension in these individuals.

Although the loss of follow-up of subjects after baseline was greater than 20%, still the eligibility rate was greater than 50%, and everything was described in detail, so this article was considered to have good methodological quality.

The study conducted by Haraldsdottir et al.^21^ was done with VLBW adults, both men and women (26–29 years). Until the present moment, it was the first study to investigate HR recovery after maximal exercise in preterm adults. Parameters for lung capacity, maximal aerobic capacity, metabolic and ventilatory parameters, HR, BP and others were collected. Two protocols were made: progressive exercises in the vertical cycle ergometer breathing the ambient air (normoxia), followed by rest and then with hypoxic air, until reaching the maximum exhaustion.

It was concluded that preterm healthy individuals with VLBW have significantly slower HR recovery when compared to those born at term, as well as lower aerobic fitness, indicating greater vulnerability to cardiovascular and metabolic diseases. Methodological quality was considered good.

The objectives of the study performed by Mathewson et al.^14^ were:

1. To verify whether HR and respiratory sinus arrhythmia (RSA) differs between adults with ELBW and NBW;
2. To verify whether the functioning of the ANS was vulnerable to age-related decline in ELBW participants. Men and women at 22–26 years old and later at 30–36 years old were analyzed. Electrocardiogram (ECG) was continuously collected in the sitting position, at rest, for two (at 22–26 years) and for six minutes (30–35 years); RSA was analyzed for five minutes; and EEG was also evaluated. RSA in individuals with ELBW was significantly lower than NBW in all evaluations, suggesting a decrease in parasympathetic regulatory control. Methodological quality was considered as good.

Jones et al.^15^ investigated small born adults (with LBW) aged approximately 26 years old. It was observed that the subjects with LBW had persistently altered ANS and baroreflex function, evaluating through stress tests. Three psychological tests were performed, each lasting five minutes each and six of them resting:

1. Color-word conflict task (the Stroop test),
2. Mirror-tracing task,
3. Speech task (hypothetical scenario of confrontation).

Information on birth, health and socioeconomic factors of childhood, current HR and BP measurements and a salivary sample were collected.

HR and BP increased in both sexes during the three tests when compared to rest; low frequency of BP variability increased at rest and during testing, high-frequency HRV levels reduced and baroreflex activity reduced in women. There was no significant difference between cardiovascular parameters in men. It has been found that impaired fetal growth, autonomic cardiovascular control and baroreflex function are related, and may be sex-dependent.

The article obtained poor methodological quality. Its objective, despite being written out in the abstract, was not clearly specified in the text; nor was the study population. There was no exact information on this sample, such as mean or minimum and maximum BW values, for example. The paper mentioned LBW, but without any numerical data. Since it did not detail the BW and other important information, the reader's interpretation may be misleading and the study is difficult to reproduce by other researchers.

The article published by Ward et al.^16^ was similar to one by Jones et al.^15^ The objective was to analyze whether younger individuals at birth had higher BP and HR responses to the same psychological stressors.

Women with higher BW had a decrease in systolic and diastolic BP in response to these stimuli, BW and HR were inversely related. There were no such relationships in men. In the conclusion, an association was made between cardiovascular reactivity, psychological stressors and size at birth.

This latter article obtained good methodological quality. The objective was very clear, as were the study population and its characteristics and the description of protocols, results, discussion and conclusion.

## DISCUSSION

In general, the main findings of the articles found and briefly described in this systematic review showed that a change in BW influences ANS in adulthood.

After investigating whether there is a relationship between BW and ANS during adulthood, we found that this subject is scarce in the literature; there are few articles published. However, it is interesting that there was a diversification in variables and types of analysis. Studies with psychological stressors, muscle sympathetic nerve path analysis, evaluation during and after physical activity, and many others made the review useful, as they expressed the relationship of the two variables studied under various stimuli.

Most publications have identified that adults born with LBW have changes in ANS. Among the nine studies presented, six (66.6%)^14-18,21^ mentioned the development of CVD or the prevalence of hypertension in adulthood; two (33.3%)^13,20^ showed no association; and four (66.6%),^13,19-21^ presented a possible risk, while three (33.3%)^14,16,18^ did not mention the subject.

The studies that did not mention these risks were from Phillips & Barker,^18^ Jones et al.^15^ and Mathewson et al.^14^ Those that did not find an association between CVD and SAH were Weitz et al.^20^ and O'Hare et al.^13^ However, despite not citing or not suggesting any risk, all papers mentioned an alteration of the ANS in some way, such as decreased autonomic control.

The latter two of the above-mentioned studies corroborated a study in five-year-old children born with LBW that presented higher sympathetic activity by van Deutekom et al.^22^ Similarly, autonomic dysfunctions were found in adolescents from the same population by Haraldsdottir et al.,^23^ which leads us to think that this autonomic dysfunction persists throughout life.

The increase in low-grade inflammation and impaired (reduced) autonomic function in young Mongolians with LBW may be a determinant for the development of SAH according to Bao et al.^19^ Along the same lines, the study performed by Ward et al.^16^ with psychological stressors also indicated the impairment of ANS through baroreflex control of cardiovascular function analysis in women with LBW, suggesting that reduced intrauterine size may result in increased chances of hypertension and further CVDs.

This may have consequences. In the “Diretrizes brasileira de hipetensão arterial–2020”,^24^ data on how hypertension is related to various cardiovascular diseases, complications and, especially, deaths were presented. This clinical condition may be associated with multifactors and can also be aggravated by other risk factors, such as dyslipidemia, glucose intolerance, diabetes *mellitus*. It may also be associated with conditions such as heart failure and chronic kidney disease. That is, individuals who have hypertension have a direct or indirect risk of developing CVD, which brings complications to life and reduces life expectancy.

The VLBW population was evaluated by Haraldsdottir et al.,^21^ analyzing HRV, lung capacity and other factors in the recovery after maximal physical activity at normoxia and hypoxia. The authors^21^ assessed preterm young adults with very low BW (<1500 g), in which recovery was assessed after maximal physical activity with normoxia and hypoxia on an ergometer cycle. Recovery of HR in the initial two minutes of both protocols was slower in preterm infants compared to full-term, which means that very normal low BW impairs autonomic function. This could indicate an increased cardiovascular disease risk. These healthy preterm newborns with VLBW showed significantly slower HR recovery, estimating a higher risk of CVD and cardiovascular mortality.

Moreover, Weitz et al.^20^ evaluated muscle sympathetic nerve activity recordings from the superficial peroneal nerve in subjects with low BW (<2500 g at term) and normal BW (3200 to 3700 g). These researchers reported lower sympathetic activity in the vasculature of muscles in healthy young people with normal low BW while HR and BP were similar when compared to young people with normal BW. According to the study, the lower sympathetic activity may have been the outcome of a change in the development of the intrauterine sympathetic ANS. Even so, the cardiovascular features may have progressively changed owing to environmental issues such as socioeconomic and/or biological factors.

Contrary to many studies, the study conducted by Perkiömäki et al.^17^ analyzed LBW, NBW and HBW. The authors identified a greater change in adults born with HBW to present lower vagal activity and baroreflex sensitivity. Thus, they emphasized that adults with HBW have a considerable cardiovascular risk later in life.

With these articles, it can be said that most are focused on adults born with LBW. Few studies aimed to investigate ELBW, VLBW and HBW. Regarding these last three variables, addressed in one study each, alterations were found in the ANS in all papers, indicating higher cardiovascular risk.

We reveal important information to the clinical research community, since cardiovascular disorders are projected to be one of the key public health challenges globally in the 21^st^ century.^25^ Moreover, impaired autonomic function is consistent with significantly higher rates of cardiovascular disease and mortality.^26,27^ Our review shows that individuals who were not born within the normal range (ELBW, VLBW, LBW and HBW) should be given extra attention from birth and throughout life, as this population may have a tendency to develop some form of CVD during the adult phase.

As a limitation of the study, we can mention the date of execution of the selection of articles. We might also add the heterogeneity of the data on birth weight (ELBW, VLBW, LBW, NBW and HBW).

In conclusion, birth weight outside the normal range may negatively influence the ANS, causing autonomic dysfunction and increasing the likelihood of cardiovascular disease and hypertension in adulthood. Thus, we emphasize the importance of monitoring by health professionals during the pregnancy and prenatal periods as well as throughout life, with preventive attention to this situation.
